# Cellular localization of the Arabidopsis class 2 phytoglobin influences somatic embryogenesis

**DOI:** 10.1093/jxb/erx003

**Published:** 2017-02-11

**Authors:** Cara Godee, Mohamed M. Mira, Owen Wally, Robert D. Hill, Claudio Stasolla

**Affiliations:** 1 Department of Plant Science, University of Manitoba, Winnipeg, Manitoba, R3T 2N2, Canada; 2 Permanent address: Department of Botany, Faculty of Science, Tanta University, Tanta, Egypt 31527; 3 Agriculture and Agri-Food Canada/Government of Canada, Harrow Research and Development Centre, RR #2, 2585 County Rd. 20, Harrow, ON N0R 1G0, Canada

**Keywords:** Auxin, dexamethasone, phytoglobin, somatic embryogenesis.

## Abstract

Mutation of phytoglobin 2 (*Pgb2*) increases the number of somatic embryos in Arabidopsis. To assess the effects of the cellular localization of Pgb2 on embryo formation, an inducible system expressing a fusion protein consisting of Pgb2 linked to the steroid-binding domain of the rat glucocorticoid receptor (GR) was introduced in a *pgb2* mutant line lacking the ability to express *Pgb2*. In this transgenic system, Pgb2 remains in the cytoplasm but migrates into the nucleus upon exposure to dexamethasone (DEX). Pgb2 retention in the cytoplasm, in the absence of DEX, increased the number of somatic embryos and reduced the expression of *MYC2* - an inhibitor of the synthesis of auxin, which is the inductive signal for embryogenesis. Removal of DEX also induced the expression of several genes involved in the biosynthesis of tryptophan and the auxin, indole-3-acetic acid (IAA). These genes included: *tryptophan synthase*-α *subunit* (*TSA1*) and *tryptophan synthase*-β *subunit* (*TSB1*), which are involved in the synthesis of tryptophan, *cytochrome P450 CYP79B2* (*CYP79B2*) and *amidase 1* (*AMI1*), which participate in the formation of IAA via indole-3-acetaldoxime, and several members of the *YUCCA* family, including *YUC1* and *4*, which are also required for IAA synthesis. Retention of Pgb2 in the cytoplasm by removal of DEX increased the staining pattern of IAA along the cotyledons of the explants generating embryogenic tissue. Staining for IAA decreased when Pgb2 translocated into the nucleus in response to the application of DEX. Collectively, these results suggest that the presence of Pgb2 in the cytoplasm, but not in the nucleus, phenocopies the effects of *Pgb2* mutation in inducing somatic embryogenesis.

## Highlight

During Arabidopsis somatic embryogenesis phytoglobin 2 exercises its effects only when targeted to the nucleus, while when cytoplasmic it mimics the effects of its mutant form.

## Introduction

Embryogenesis is a crucial event in the plant life cycle. It is initiated by the formation of the zygote, which through precise and conserved cell division and differentiation patterns, generates a mature embryo consisting of an embryonic axis separating the shoot and root apical meristems and one or more cotyledons (Jenik *et al.* 2007). The embryogenic program can be recapitulated *in vitro* through manipulating the media and culture components that induce cells to reprogram their developmental fate and embark along an embryogenic pathway. The genetic basis of this reprogramming is largely unknown (Karami *et al.* 2009). This process is best exemplified during somatic embryogenesis where somatic cells, i.e. cells other than gametes, can produce genetically identical somatic embryos with structural and physiological features resembling those of their zygotic counterparts (Stasolla and Yeung, 2003). These desirable characteristics, as well as the possibility to generate a large number of synchronous embryos in an enclosed environment and in a short period of time, make somatic embryogenesis a suitable model to investigate the structural, physiological and molecular events governing embryogenesis (Mordhorst *et al.* 2002; Su and Zhang 2009).

In Arabidopsis, somatic embryogenesis can be initiated by culturing cotyledonary zygotic embryos on an induction medium containing auxin, which is the inductive signal required for the dedifferentiation and redirection of somatic cells towards the embryogenic route (Raghavan, 2004). The initial phases on induction medium involve the expansion of the cotyledons followed by the generation of embryogenic tissue arising from the adaxial side of the cotyledons. Removal of exogenous auxin triggers the development of the somatic embryo (see Supplementary Fig. S1 available at *JXB* online). Formation of the embryogenic tissue during the induction phase is mainly the result of localized proliferation along the surface of the cotyledons, as the remaining regions of the zygotic embryos do not form embryogenic cells (Raghavan, 2004). Generation of auxin maxima, through PIN1-mediated polar transport, along the embryogenic tissue is essential for the establishment of stem cells and the generation of somatic embryos (Su and Zhang, 2009). Factors influencing these processes have begun to emerge (Bai *et al.*, 2013; Elhiti *et al.*, 2013).

Hemoglobins are present in all nucleated organisms and have been well characterized for their ability to bind and transport oxygen and other small gas ligands. In plants, proteins with hemoglobin-like features were initially found in nodules containing nitrogen-fixing bacteria and named leghemoglobins (Smagghe *et al.*, 2009), while those later associated with organs other than nodules were referred as non-symbiotic hemoglobins (Hunt *et al.*, 2001). The subsequent characterization of several types of plant hemoglobins with diverse functions (Garrocho-Villegas *et al.*, 2007; Hill, 2012) has resulted in a change in nomenclature from plant hemoglobins to phytoglobins (Pgbs) (Hill *et al.*, 2016).

Three classes of Pgbs with distinct oxygen binding characteristics and expression patterns have been described (Hunt *et al.*, 2001), with the majority of studies centered around members of class 1 and 2. The two classes mainly differ in their oxygen binding affinities with a Km close to 2 nM for members of class 1 and 150 nM for class 2 (Hoy and Hargrove, 2008; Dordas, 2009). The Arabidopsis class 1 Pgb, Pgb1, has been mainly studied in relation to its capacity to scavenge nitric oxide (NO) in low oxygen environments (Dordas *et al.*, 2003; Dordas *et al.*, 2004), and its ability to protect plant cells during prolonged stress conditions (Perazzolli *et al.*, 2004). This protective role has been further confirmed by the increase in survival and maintenance of the energy status observed in Arabidopsis roots overexpressing *Pgb1* (Hunt *et al.*, 2002). While less is known about the Arabidopsis class 2 Pgb, Pgb2, its involvement in stress responses through mechanisms characteristic of Pgb1 cannot be discounted. Like *Pgb1*, overexpression of *Pgb2* enhances survival under hypoxic conditions through removal of cellular NO (Hebelstrup *et al.*, 2006; Hebelstrup and Jensen, 2008; Hebelstrup *et al.*, 2012). Distinct conditions inducing the two Pgbs, with cytokinin and cold temperatures only effective in increasing expression of *Pgb2*, suggest that the functions of Pgb1 and Pgb2 might not be fully redundant. This notion is further confirmed by their different localization patterns, with Pgb2 preferentially expressed in young organs including immature seeds, fruit, and leaves, as well as somatic embryos (Hendriks *et al.*, 1998; Hunt *et al.*, 2002; Wang *et al.*, 2003).

Deviation in morphogenesis occurs as a result of altered *Pgb2* expression. Besides affecting meristem function by accelerating the transition of vegetative meristems into inflorescence meristems (Hebelstrup and Jensen, 2008) and enhancing *in vitro* shoot organogenesis when ectopically expressed (Wang *et al.*, 2011), Pgb2 regulates Arabidopsis somatic embryogenesis. Mutation of *Pgb2* increases the number of Arabidopsis somatic embryos by suppressing the expression of *MYC2*, a repressor of auxin synthesis, and inducing transcription of several indole-3-acetic acid (IAA) biosynthetic genes (Elhiti *et al.*, 2013). These transcriptional changes resulted in elevated levels of IAA within the embryogenic tissue generated from the adaxial sides of the cotyledons of the explants (Elhiti *et al.*, 2013). Generation of IAA maxima, mediated by relocation of PIN1, marks the future somatic embryo initiation sites and encourages stem cell formation and production of somatic embryos (Su and Zhang, 2009; Elhiti *et al.*, 2013). The precise transduction pathway triggered by mutation of *Pgb2* and resulting in enhanced IAA production has yet to be determined and the responses controlled by Pgb2 might be influenced by its cellular localization. Phytoglobins lack specific organelle localization signals although independent reports demonstrate their presence in the cytosol and nucleus. The alfalfa Pgb protein, Mhpgb1, was localized mainly in the nucleus with only small traces detected in the cytoplasm (Seregélyes *et al.*, 2000); an observation subsequently confirmed in Arabidopsis (Hebelstrup *et al.*, 2008). Two mammalian hemoglobins, neuroglobin and cytoglobin, have also been detected in the nucleus (Geuens *et al.*, 2003; Hundahl *et al.*, 2010). Whether cellular localization i.e. cytoplasm versus nucleus, determines the Pgb response needs to be clarified.

To further understand the mode of action of Pgb2 during embryogenesis and to determine the implications of its cellular localization on the observed response, an inducible system expressing a fusion between Pgb2 and the steroid-binding domain of the rat glucocorticoid receptor (GR) was introduced in a *Pgb2* mutant background. This system, in which the Pgb2-GR fusion protein remains in the cytoplasm but migrates into the nucleus upon exposure to dexamethasone (DEX), was used during Arabidopsis somatic embryogenesis to demonstrate that Pgb2 function occurs only when the protein is present in the nucleus. Retention of Pgb2 in the cytoplasm mimics *pgb2* mutant phenotypes: it promotes the transcriptional activation of IAA biosynthetic genes, favors the accumulation of IAA within embryogenic tissue arising from the zygotic embryo explants, and enhances the formation of somatic embryos.

## Materials and methods

### Generation of the Pgb2 lines

The 35S:Pgb2-GR (A) and (B) lines were produced by introducing the Pgb2-GR fusion protein, driven by a 35S promoter, into *pgb2* mutant plants. The lines were generated by subcloning *Pgb2* from Arabidopsis cDNA and adding the attB1 and attB2 sites to the 5’ and 3’ ends removing the stop codon using the following primers: attb1-F adaptor, attb2-R adaptor, AtHB2-F attb1, and AtHB2-R no stop (Supplementary Table S1). By using BP clonase (Invitrogen), the fragment was then placed in the BP cloning vector pDONR Zeo to create pENTR Pgb2 zeo. The system was used to transfer the *Pgb2* fragment into the R1R2 sites of R1R2 GR vector (Baud *et al.*, 2007), using Gateway® LR Clonase® II Enzyme mix (Thermo Fisher Scientific). The resulting R1R2 GR Pgb2 vector was then employed to transform *pgb2* plants containing a T-DNA insert disrupting *Pgb2,* (Hebelstrup *et al.*, 2006) using GV3101 *Agrobacterium tumefaciens* via floral dipping (Clough and Bent, 1998). Selection was performed by germinating the seeds on 1/2 Murashige Skooge (MS) medium (Murashige and Skooge, 1962) containing 25 mg/L kanamycin. Plants were then screened to detect the presence of the construct using the 35S-F and Pgb2-R primers, as well as the presence of the T-DNA insert using the Pgb2-F and dSmp-R primers (see primers list in Supplementary Table S1). Plants from the T3 generation were utilized for this study.

### Transient transformation of Arabidopsis seedlings

Transient expression of Arabidopsis seedlings was used to verify the targeting of Pgb2 into the nucleus following exposure to DEX. The YFP-Pgb2 fusion construct was cloned into the same vector R1R2 GR used for stable transformation. Generation of the YFP-Pgb2 fusion construct is briefly described below. The gBlock fragment SalI-YFP-linker-Pgb2-SalI (Supplementary Fig. S2), custom synthesized by Integrated DNA Technology, was digested with the SalI restriction enzyme. The fragment was subsequently ligated with a SalI-digested Gateway® pENTR^TM^1A Dual Selection Vector (Thermo Fisher Scientific) according to the manufacturer’s instructions. The orientation of the inserted cassette was verified using YFB-F and Pentr1A-R primers. The YFP-Pgb2 fusion construct was cloned into the R1R2 GR vector using the Gateway® LR Clonase® II Enzyme mix (Thermo Fisher Scientific) and then transformed into GV3101 *A. tumefaciens* cells. Transient transformation of Arabidopsis seedlings was conducted exactly as reported by Li *et al.* (2009). DEX at a concentration of 20 μM was applied 3 days prior to Agrobacterium cocultivation, which occurred seven days after germination. Pgb2 was visualized on leaf cells using confocal microscopy.

### Generation of Arabidopsis somatic embryos

Arabidopsis seeds were sterilized using 70% ethanol, 0.5% Triton X100 for 15 minutes followed by 95% ethanol for 15 minutes. The seeds were then transferred on ½ strength MS medium, incubated at 4 °C in the dark for 2–3 days and then transferred to a growth cabinet at a temperature of 20–22 °C, with a 16 h light/8 h dark photoperiod. Plants were grown and green siliques were used for dissection of immature zygotic embryos.

Somatic embryogenesis was initiated according to Bassuner *et al.* (2007). Immature zygotic embryos were plated on induction medium containing 2,4-dichlorophenoxyacetic acid (2,4-D) for 14 days, followed by a transfer onto hormone-free development medium. Efficiency of somatic embryogenesis was estimated by counting the number of fully developed somatic embryos after 9 days on development medium. Each biological replicate consisted of one petri plate containing 20 zygotic explants and three biological replicates were used for each measurement.

### Chemical treatments

The NO scavenger 2-(4-carboxyphenyl)-4,4,5,5-tetramethylimidazoline-1-oxyl-3-oxide (cPTIO) was applied as specified in Elhiti *et al.* (2013), by dispensing 10 μl of a 10 µM solution directly on the explants every other day in culture throughout the induction medium. DEX was dissolved in 20% ethanol and added to induction medium to a final concentration of 20 µM.

### Analyses of transcript levels

Total RNA was extracted from day 7 and day 14 explants on induction medium using TRI Reagent (Invitrogen) followed by a treatment with DNase I (RNase-free, Promega). The RNA was then utilized for the synthesis of cDNA using the High Capacity cDNA Reverse Transcription Kit (Applied Biosystems). Quantitative real-time PCR was performed exactly as documented by Elhiti *et al.* (2013) using primers listed in Supplementary Table S1. The relative level of gene expression was analyzed by the 2^−∆∆Ct^ method described by Livak and Schmittgen (2001), using UBQ10 (AT4G05320) as a reference (Czechowski *et al.*, 2005; Hong *et al.*, 2010).

### Localization of IAA

Immunolocalization of endogenous IAA was performed exactly as described by Mira *et al.* (2016). Explants at day 7 on induction medium were pre-fixed in 4% aqueous 1-ethyl-3-(3-dimethyl-aminopropyl)-carbodiimide hydrochloride at 4 °C for 2 h, post-fixed in FAA (10% formalin, 5% acetic acid, and 50% ethanol) overnight at 4 °C, dehydrated in an ethanol series, and embedded in Paraplast Plus (Fisher). The tissue was then sectioned to a thickness of 10 µm and deparaffinised in xylene. The sections were incubated in blocking solution [1 x phosphate-buffered saline (PBS) solution pH 7, 0.1% Tween 20, 1.5% glycine, and 5% bovine serum albumin (BSA)] for 1 hour. 150 µl of monoclonal primary IAA-antibodies (1 mg/ml, Sigma) diluted 1:200 in 1x PBS containing 0.8% BSA were applied to the sections and incubated in a high humidity chamber for 4 hours at room temperature. The slides were washed first in 1x PBS containing 0.88 g/L NaCl, 0.1% Tween 20, and 0.8% BSA for 5 minutes and then in 1xPBS with 0.8% BSA for 5 minutes in order to remove any excess Tween 20. The slides were incubated in 200 µl of secondary antibody [anti-mouse IgG alkaline phosphatase conjugate (1 mg/ml), Promega, USA] overnight in a high humidity chamber, washed two times in 1x PBS containing 0.88 g/L NaCl, 0.1% Tween 20, and 0.8% BSA for 10 minutes and then incubated in water for 15 minutes to remove the excess secondary antibodies. Samples were stained using 250 µl Western Blue Stabilized Substrate for Alkaline Phosphatase (Promega) for 40 minutes.

### RNA *in situ* hybridization

RNA *in situ* hybridization studies were performed according to Elhiti *et al.* (2010). The two full length *YUC1* and *YUC4* cDNAs were cloned into a pGEM-T Easy Vector System (Promega). The two cDNAs were subsequently amplified from the vector using T7 and SP6 primers and used for the preparation of digoxigenin (DIG)-labelled sense and antisense riboprobes, following the procedure outlined in the DIG Application Manual (Roche Diagnostics). Slide preparation, hybridization conditions, and color development were performed as documented previously (Elhiti *et al.*, 2010).

### Statistical analyses

The cDNA of at least three biological replicates was used for quantitative RT-PCR studies. Differences in relative transcript levels were analyzed by comparing the Least Squares (LS) Means of the interaction effect, with or without DEX treatment on genotype, using Tukey’s test. The SAS® 9.3 program (SAS Institute Inc., Cary, NC, USA) was used for all statistical analyses.

## Results

### Cellular localization of Pgb2 influences Arabidopsis somatic embryogenesis

**Fig. 1.**
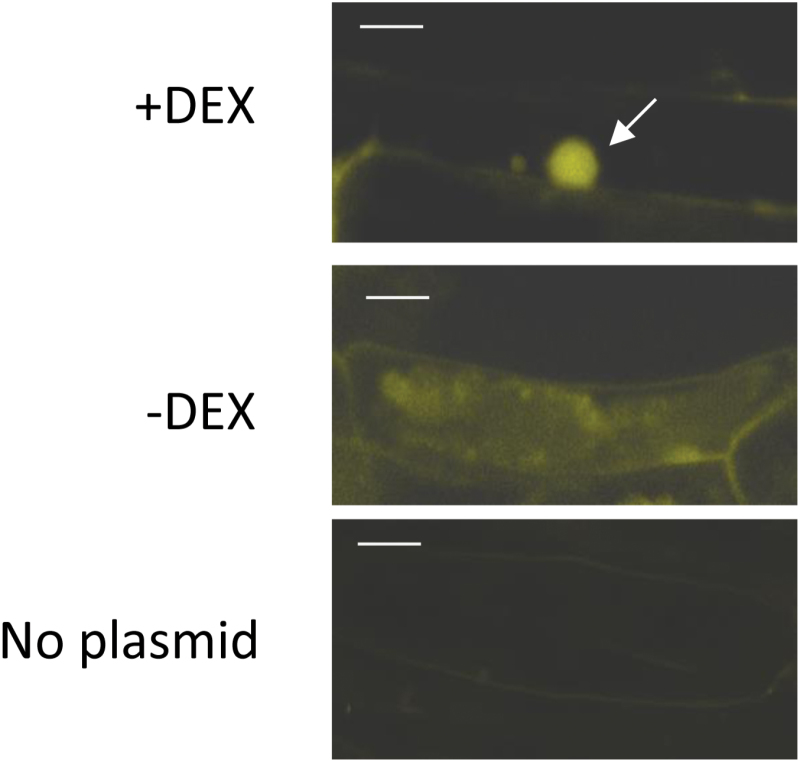
Transient transformation of Arabidopsis seedlings with 35S:Pgb2-GR-YFP. Addition of DEX targets Pgb2 to the nucleus (arrow), while in the absence of DEX Pgb2 is retained in the cytoplasm. Scale bars, 5 μm.

To evaluate how cellular localization of Pgb2 affects somatic embryogenesis we generated an Arabidopsis line expressing a fusion protein consisting of Pgb2 linked to the steroid-binding domain of the rat glucocorticoid receptor (GR) (Lloyd *et al.*, 1994). The expressed PGB2-GR fusion protein remains in the cytoplasm but can be translocated into the nucleus when the steroid dexamethasone (DEX) is bound to the GR receptor site. The effectiveness of the system was tested in Arabidopsis seedlings using transient expression experiments in which the application of DEX was sufficient to translocate PGB2-YFP-GR into the nucleus (Fig. 1).

The 35S:Pgb2-GR construct was introduced in the *pgb2* Arabidopsis mutant and two independent transformed lines, 35S:Pgb2-GR (A) and (B), were generated (Supplementary Fig. S3). The behavior of both lines during somatic embryogenesis was then compared to a line ectopically expressing *Pgb2*, 35S:Pgb2 line, and a line in which *Pgb2* was mutated, *pgb2* line. These lines have been characterized in previous studies (Hebelstrup *et al.*, 2006; Elhiti *et al.*, 2013).

**Fig. 2.**
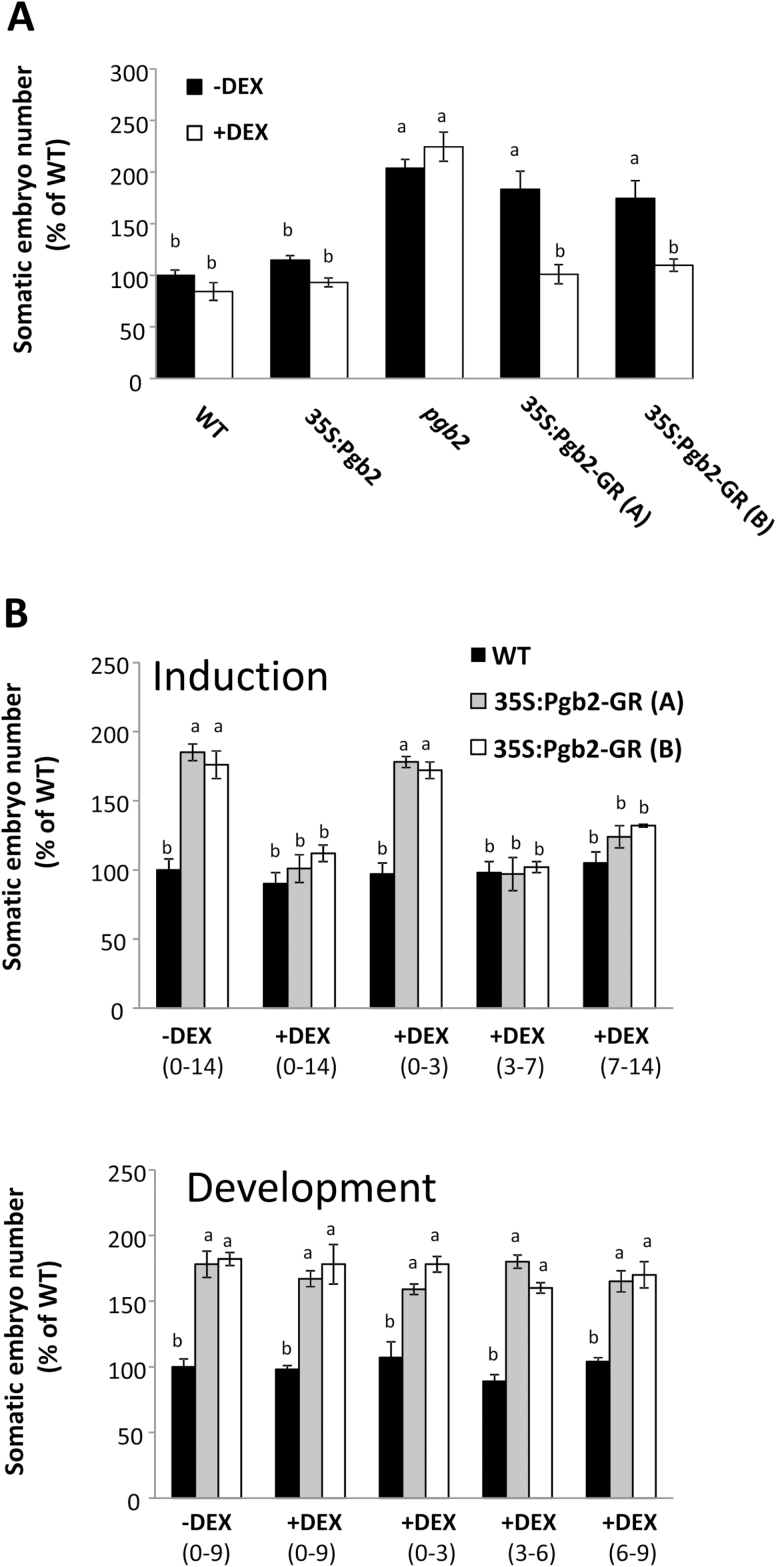
Effects of Pgb2 on somatic embryogenesis. **(A)** Number of Arabidopsis somatic embryos produced by the different lines. Values±SE are means of at least three biological replicates and are normalized to the value of wild type without DEX set at 100%. Letters on bars indicate statistically significant differences, *P*≤0.05. WT, wild type line; 35S:Pgb2, line ectopically expressing *Pgb2*; *pgb2*, line suppressing *Pgb2*; 35S:Pgb2-GR (A) and (B), lines in which Pgb2 tagged to the glucocorticoid receptor (GR) was overexpressed in a *pgb2* background. **(B)** Applications of DEX at different days in culture during induction and development of the 35S:Pgb2-GR (A) and (B) lines. Values±SE are means of at least three biological replicates and are normalized to the value of wild type without DEX during days 0–14 for induction and wild type without DEX during days 0–9 for development set at 100%. Letters on bars indicate statistically significant differences, *P*≤0.05.

Relative to wild type, the number of embryos was not affected in the 35S:Pgb2 line while it increased in the *pgb2* line, independently from DEX (Fig. 2A). An increase of similar magnitude was also observed in the 35S:Pgb2-GR (A) and (B) lines in the absence of DEX, while addition of DEX in the induction medium reduced embryo production to wild type values (Fig. 2A).

To estimate the temporal requirement of Pgb2 compartmentalization for somatic embryo formation, DEX was applied to the 35S:Pgb2-GR lines at different intervals during induction or development. In the wild type line applications of DEX at different days of induction had no significant effects on embryo yield (Fig. 2B). This was in contrast to the 35S:Pgb2-GR lines where inclusions of DEX during the first three days resulted in somatic embryo values similar to those measured in the absence of DEX. Applications of DEX after day 3 in the induction phase reduced embryo values to those of the wild type (Fig. 2B). Addition of DEX at any stage during embryo development had no effect on embryo number, producing embryo numbers similar to values in the absence of DEX.

**Fig. 3.**
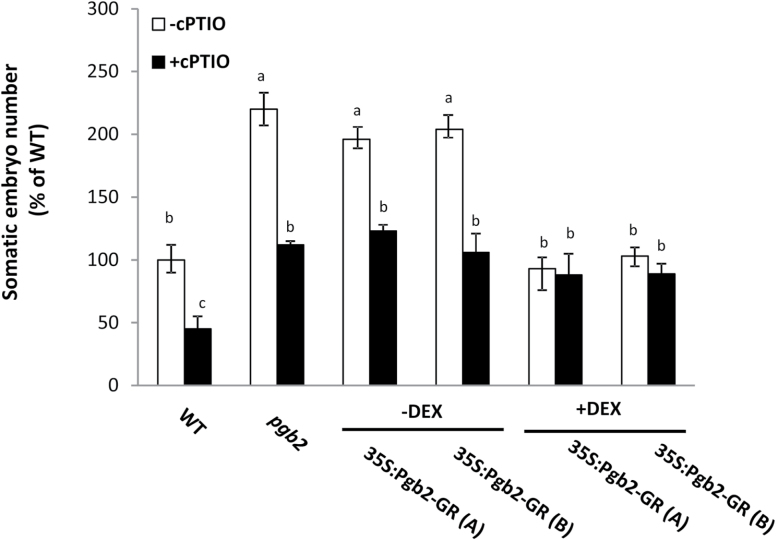
Number of somatic embryos produced by the same lines used in Fig. 2 in the presence or absence of the nitric oxide scavanger cPTIO added during the induction period. Values±SE are means of at least three biological replicates and are normalized to the value of wild type without cPTIO set at 100%. Letters on bars indicate statistically significant differences, *P*≤0.05.

An established function of Pgbs is to scavenge nitric oxide (NO) (Hill, 2012). An experimental reduction of NO through pharmacological treatments reverses the effects of *Pgb2* suppression on somatic embryogenesis (Elhiti *et al.*, 2013). To evaluate if the observed influence of Pgb2 compartmentalization on embryo production is dependent on NO, the NO scavenger cPTIO was added to the induction medium. Consistent with previous studies (Elhiti *et al.*, 2013), cPTIO reduced embryo production in both wild type and *pgb2* lines (Fig. 3). A similar cPTIO repressive effect was also observed in the two 35S:Pgb2-GR (A) and (B) lines in the absence of DEX, but not in the same lines cultured with DEX (Fig. 3).

Collectively, these results suggest that: 1) somatic embryo production is enhanced in the *pgb2* mutant and this phenotype can be reversed by the re-introduction of Pgb2 in the nucleus but not in the cytoplasm; 2) the promotive effect on embryogenesis of suppression of *Pgb2* or its restriction to the cytoplasm, occurs after day 3 on induction medium in conjunction with the formation of embryogenic tissue; and 3) this promotive effect can be attenuated by reducing the level of NO.

### Transcription of *MYC2* and genes of the indole acetic acid (IAA) biosynthetic pathway is affected by the cellular localization of Pgb2

Auxin is the inductive signal required for the initiation of somatic embryogenesis (Raghavan, 2004). By suppressing *MYC2*, mutation of *Pgb2* induces the IAA biosynthetic machinery and generates IAA maxima within the embryogenic tissue arising from the adaxial side of the cotyledons of the explants (Elhiti *et al.*, 2013). To determine if these events are influenced by the cellular location of Pgb2, the transcript levels of MYC2 and several genes encoding auxin biosynthetic enzymes, which are upregulated by mutation of *Pgb2* (Elhiti *et al.*, 2013), were measured at day 7 and 14 on induction medium.

**Fig. 4.**
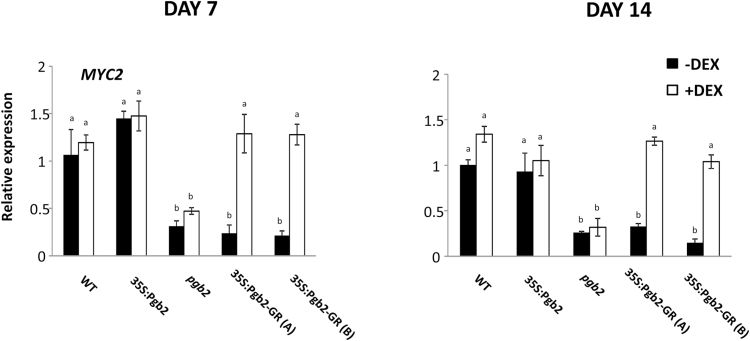
Relative transcript levels of *MYC2* at day 7 and 14 of induction. Values±SE are means of three biological replicates and are normalized to the value of wild type without DEX set at 1. Letters on bars indicate statistically significant differences, *P*≤0.05. Lines utilized: WT, wild type line; 35S:Pgb2, line ectopically expressing *Pgb2*; *pgb2*, line suppressing *Pgb2*; 35S:Pgb2-GR (A) and (B), lines in which Pgb2 tagged to the glucocorticoid receptor (GR) was overexpressed in a *pgb2* background.

**Fig. 5.**
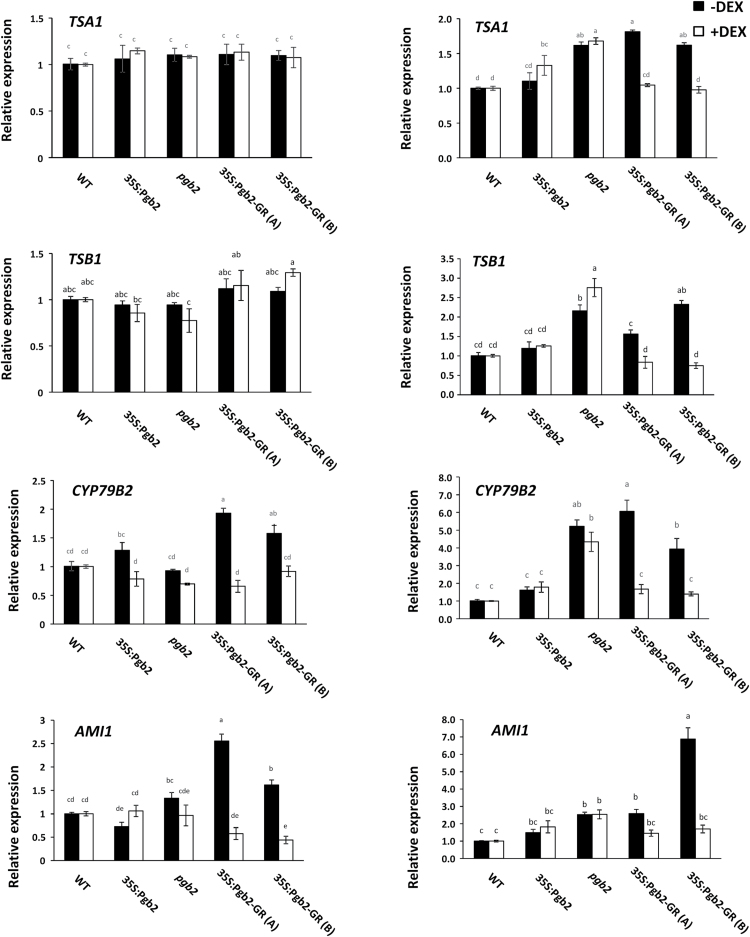
Relative transcript levels of genes participating in auxin biosynthesis at day 7 and 14 of induction. Values±SE are means of three biological replicates and are normalized to the respective wild type values, with or without DEX, set at 1. Letters on bars indicate statistically significant differences, *P*≤0.05. Genes measured included: *tryptophan synthase*-α *subunit* (*TSA1*)*, tryptophan synthase*-β *subunit* (*TSB1*)*, cytochrome P450 CYP79B2* (*CYP79B2*) *and amidase 1* (*AMI1*). Lines utilized: WT, wild type line; 35S:Pgb2, line ectopically expressing *Pgb2*; *pgb2*, line suppressing *Pgb2*; 35S:Pgb2-GR (A) and (B), lines in which Pgb2 tagged to the glucocorticoid receptor (GR) was overexpressed in a *pgb2* background.

Relative to wild type, expression of *MYC2* was reduced in the *pgb2* mutant line and in the two inducible 35S:Pgb2-GR (A) and (B) lines cultured in the absence of DEX (Fig. 4). Applications of DEX in the inducible lines increased the transcript levels of *MYC2*. Genes involved in the last step of tryptophan synthesis, *tryptophan synthase*-α *subunit* (*TSA1*) and *tryptophan synthase*-β *subunit* (*TSB1*), were not affected by altered levels of *Pgb2* at day 7. However, at the end of the induction period the expression of both genes increased in the *pgb2* line independently from DEX, and in the 35S:Pgb2-GR (A) and (B) lines in the absence of DEX (Fig. 5).

**Fig. 6.**
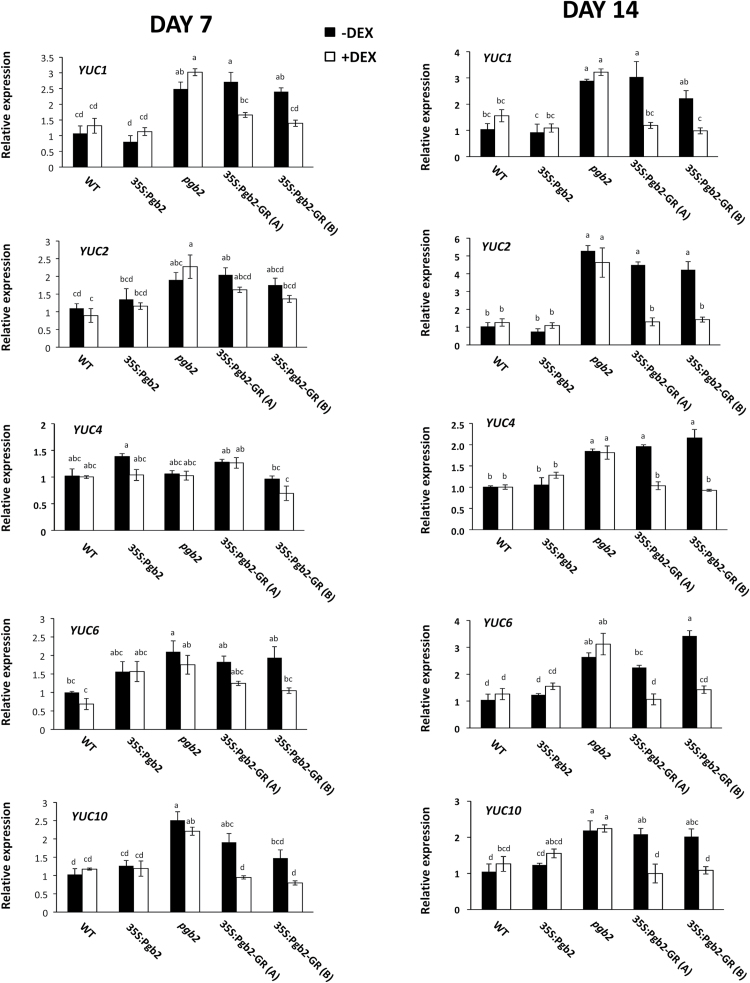
Relative transcript levels of *YUCCA 1, 2, 4, 6* and *10* at day 7 and 14 of induction. Values±SE are means of three biological replicates and are normalized to the respective wild type values, with or without DEX, set at 1. Letters on bars indicate statistically significant differences, *P*≤0.05. Lines utilized: WT, wild type line; 35S:Pgb2, line ectopically expressing *Pgb2*; *pgb2*, line suppressing *Pgb2*; 35S:Pgb2-GR (A) and (B), lines in which Pgb2 tagged to the glucocorticoid receptor (GR) was overexpressed in a *pgb2* background.

In Arabidopsis, conversion of tryptophan to IAA can occur through three distinct routes (Mashiguchi *et al.*, 2011). The first is regulated by the sequential activity of cytochrome P450 CYP79B2 (CYP79B2) and amidase 1 (AMI1), the former producing indole-3-acetaldoxime from tryptophan and the latter converting indole-3-acetaldoxime to IAA. Conversion of tryptophan to IAA can also occur through a poorly characterized second route or through the YUCCA pathway involving members of the YUCCA family. Relative to wild type, the expression of both *CYP79B2* and *AMI1* increased in the two 35S:Pgb2-GR (A) and (B) lines cultured without DEX at both day 7 and 14 on induction medium (Fig. 5). In the same lines, application of DEX reduced the expression of both genes to wild type values. A DEX independent increase in CYP79B2 transcript levels was also observed in the *pgb2* explants at the end of the induction period. The transcript levels of the most representative *YUCCA* genes operating during Arabidopsis embryogenesis, *YUC1, 2, 4, 6,* and *10,* were also measured during the induction period. At day 7, with the exception of *YUC4*, all *YUCs* were induced in the *pgb2* line and in the 35S:Pgb2-GR (A) and (B) lines cultured in the absence of DEX (Fig. 6). A similar profile was also observed for all *YUCs* at day 14 (Fig. 6). Previous studies showed that *YUC1* and *4* can be used as markers for future somatic embryo initiation sites in explants (Bai *et al.*, 2013). To assess if exclusion of Pgb2 from the nucleus increases the domains of these sites, the transcripts of both genes were localized in explants of the 35S:Pgb2-GR (A) and (B) lines cultured in the presence or absence of DEX. In media devoid of DEX the distribution of both YUC1 and 4 transcripts extended throughout the embryogenic tissue (arrows, Supplementary Fig. S4), while only a very faint signal was observed in the embryogenic tissue of the same lines cultured with DEX (Supplementary Fig. S4).

### Suppression of *Pgb2* or retention of Pgb2 in the cytoplasm increases IAA signal in the embryogenic tissue

**Fig. 7.**
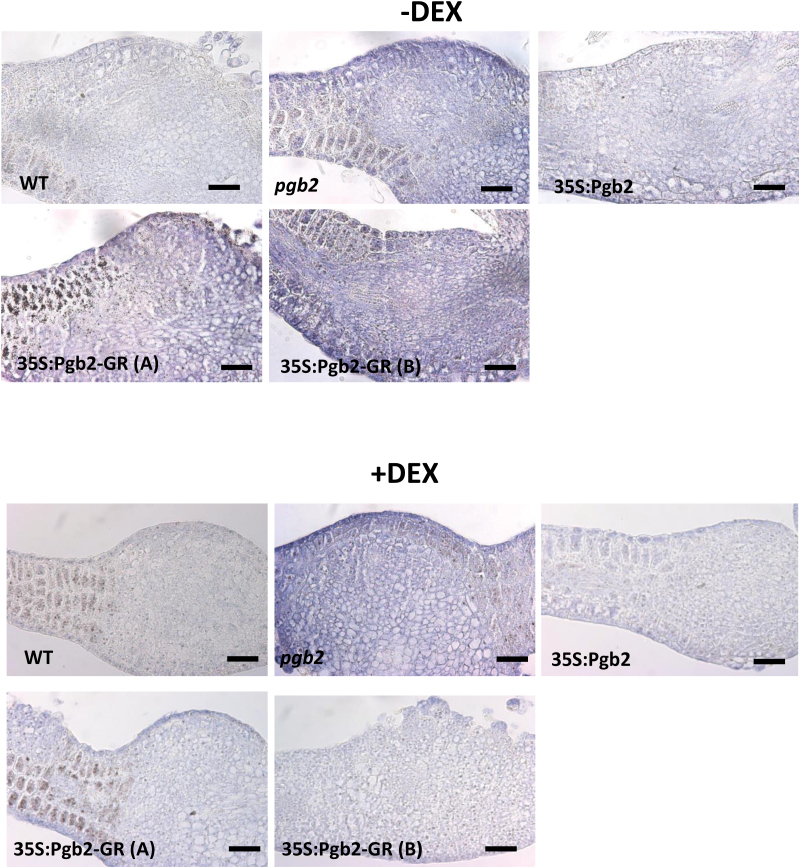
Immunolocalization of IAA at day 7 of induction in expanded cotyledons of explants of the wild type (WT) line, the 35S:Pgb2 line ectopically expressing *Pgb2,* the *pgb2* line suppressing *Pgb2*, and the 35S:Pgb2-GR (A) and (B) lines in which Pgb2 tagged to the glucocorticoid receptor (GR) was overexpressed in a *pgb2* background. Lines were cultured in the presence or absence of DEX. Scale bars, 75 μm.

Production of somatic embryos is preceded by a heavy accumulation of IAA within the embryogenic tissue arising from the cotyledons of the zygotic explants (Elhiti *et al.*, 2013). Immunocalization studies in all Arabidopsis lines were conducted to detect IAA on the adaxial sides of the cotyledons of the zygotic embryo explants; these are the sites where embryogenic tissue forms. IAA accumulated along the cotyledons of the *pgb2* explants (Fig. 7), an observation consistent with previous studies (Elhiti *et al.*, 2013). The IAA signal was weak in wild type and 35S:Pgb2 explants independently from DEX, but intense in both 35S:Pgb2-GR (A) and (B) lines cultured in the absence of DEX. Inclusion of DEX in the same lines reduced the IAA signal (Fig. 7).

Establishment of IAA maxima during the induction of Arabidopsis somatic embryogenesis is mediated by PIN1 (Su and Zhang, 2009), the expression of which is induced in highly embryogenic systems (Elhiti *et al.*, 2013). Relative to wild type and 35S:Pgb2 explants, *PIN1* transcript levels are induced at both day 7 and 14 in the *pgb2* explants and in explants of the two 35S:Pgb2-GR (A) and (B) lines cultured without DEX (Supplementary Fig. S5).

Although the mode of action of auxin in the promotion of embryogenic tissue is debatable, accumulation of IAA increases the expression of genes directing the progression of somatic embryogenesis (Raghavan, 2004). The most representative example is *somatic embryogenesis receptor kinase 1* (*SERK1*) (Supplementary Fig. S6), a gene sufficient to induce embryogenic competence in culture by encouraging the somatic-embryogenic cell fate transition (Hecht *et al.*, 2001; Kwaaitaal and De Vries, 2007). Consistent with auxin, at the end of the induction period the relative levels of *SERK1* transcripts are elevated in explants of the *pgb2* line and in those of the 35S:Pgb2-GR lines cultured in the absence of DEX. Inclusion of DEX in the 35S:Pgb2-GR lines reduced the expression of *SERK1* to levels comparable to the wild type and 35S:Pgb2 lines (Supplementary Fig. S6).

## Discussion

Arabidopsis somatic embryogenesis consists of two distinct phases: an induction phase resulting in the formation of the embryogenic tissue from the adaxial side of the cotyledons of the zygotic embryo explants and an auxin-free development phase culminating with the formation of fully developed somatic embryos. It is during the induction phase that the explants start undergoing profound morphological changes, involving a cell expansion phase (days 0–3) followed by a proliferation phase (days 3–14) producing embryogenic tissue (Raghavan, 2004; Supplementary Fig. S1). These events are limited to the cotyledons of the explants as other regions of the cultured zygotic embryos do not contribute to the formation of the embryogenic tissue (Raghavan, 2004) and are tightly regulated by unknown auxin-mediated mechanisms. Besides promoting the somatic-embryogenic transition, auxin accumulates in regions of the embryogenic tissue that will give rise to somatic embryos (Su and Zhang, 2009). Genetic or pharmacological perturbations dissipating auxin accumulation inhibit the formation of the somatic embryos (Su and Zhang, 2009). The requirement of auxin for the production of somatic embryos is not restricted to Arabidopsis and is documented in a large number of species (Thorpe and Stasolla, 2001).

Auxin responses are tightly regulated by cellular NO (Pagnussat *et al.*, 2003) and fluctuations in NO homeostasis have been implicated in many plant defense and stress response mechanisms (Delledonne *et al.*, 1998; Mackerness *et al.*, 2001; Gould *et al.*, 2003), as well as morphogenic events (Leshem and Haramaty, 1996; Beligni and Lamattina, 2000; Seregelyes *et al.*, 2003). Initially identified in the regulation of post-embryonic processes, such as root hair development, lateral and adventitious root formation, root gravitropic bending and root responses to iron deficiency (reviewed by Hill, 2012), the link between NO and auxin has been more recently extended to embryogenesis. During alfalfa somatic embryogenesis, NO is required in the induction phase where it promotes the auxin-mediated activation of cell division and embryogenic cell formation (Otvos *et al.*, 2005). Similarly, a rise in NO levels triggered by mutation of Arabidopsis *Pgb2*, increases somatic embryogenesis through suppression of *MYC2* (Elhiti *et al.*, 2013). Suppression of *MYC2*, a repressor of IAA synthesis, induces the expression of several IAA biosynthetic enzymes and alters the pattern of IAA distribution, possibly by affecting the auxin transporter, PIN1 (Elhiti *et al.*, 2013).

While the main role assigned to Pgbs is to scavenge NO produced as a result of using nitrite as an alternative electron acceptor, Igamberdiev and Hill (2004) did not exclude other physiological roles based on the presence of Pgbs in the nucleus, as demonstrated in alfalfa (Seregélyes *et al.*, 2000) and Arabidopsis (Hebelstrup *et al.*, 2008). These observations, also consistent with the localization patterns of the mamalian hemoglobins, cytoglobin and neuroglobin (Geuens *et al.*, 2003; Hundahl *et al.*, 2010), do not however prove whether the nuclear localization of Pgbs is required to produce an effect.

Using Arabidopsis somatic embryogenesis as a model system, we demonstrate that the Pgb2 response is triggered only when the protein is present in the nucleus. The enhanced embryo yield in the *Pgb2* mutant line, *pgb2* line, is phenocopied when Pgb2 is retained in the cytoplasm i.e. 35S:Pgb2-GR (A) and (B) lines cultured in the absence of DEX, and reverted to wild type values when Pgb2 is transported to the nucleus as a result of the binding of DEX to Pgb2-GR in the 35S:Pgb2-GR (A) and (B) lines (Fig. 2). This reversion is limited to a specific window of time of day, namely 3–14 on induction medium (Fig. 2B), which coincides with the formation of the embryogenic tissue (Supplementary Fig. S1). The level of NO influences the Pgb2 response (Fig. 3), in agreement with the well-established role of Pgbs as effective NO scavangers (Igamberdiev and Hill, 2004).

Formation of embryogenic tissue is closely linked to production of IAA (Raghavan, 2004; Su and Zhang, 2009), which is stimulated by suppression of *MYC2* (Elhiti *et al.*, 2013). The transcript levels of this gene are reduced by mutation of *Pgb2* or when the protein is retained in the cytoplasm but are increased when Pgb2 is translocated into the nucleus (Fig. 4). Transcription of this gene influences the synthesis of IAA (Elhiti *et al.*, 2013), which in Arabidopsis is mainly initiated by tryptophan though three distinct pathways (Mashiguchi *et al.*, 2011). While the indole-3-acetaldoxime pathway requires the enzyme CYP79B, which converts tryptophan to indole-3-acetaldoxime, and AMI1 producing IAA from indole-3-acetaldoxime, the second pathway generates indole-3-acetaldoxime through unknown mechanisms. The third route of IAA synthesis is the YUCCA pathway that involves a variety of members of the YUCCA family (Mashiguchi *et al.*, 2011). Confirming previous studies (Elhiti *et al.*, 2013), suppression of *Pgb2* enhances the expression of key tryptophan biosynthetic enzymes, such as *TSA1* and *TSB1*, and this induction is only observed in those situations where Pgb2 is excluded from the nucleus i.e. *pgb2* line and 35S:Pgb2-GR (A) and (B) lines cultured in the absence of DEX. A similar requirement was also observed for the induction of *CYP79B2* and *AMI1*, regulating the indole-3-acetaldoxime pathway, as well as *YUC1, 2, 4, 6,* and *10,* the major key players of the YUCCA pathway (Mashiguchi *et al.*, 2011). The consistent induction of *YUCs* at both day 7and 14, triggered by mutation of *Pgb2* or by its exclusion from the nucleus (Fig. 2A), is in agreement with the requirement of *YUC* genes for the formation of embryogenic tissue and the initiation of somatic embryogenesis (Bai *et al.*, 2013). Consistent with this concept, staining of *YUC1* and *4* transcripts, accurate markers of somatic embryo initiation sites (Bai *et al.*, 2013), extended throughout the embryogenic tissue in situations when Pgb2 was excluded from the nucleus (Supplementary Fig. S4).

The transcriptional induction of tryptophan and IAA biosynthetic genes observed in the *pgb2* line and 35S:Pgb2-GR (A) and (B) lines cultured in the absence of DEX coincides with the intense IAA staining pattern throughout the embryogenic tissue arising from the cotyledons of the zygotic explants (Fig. 7). We have previously demonstrated that this preferential accumulation of IAA in cotyledonary cells is also mediated by the auxin transporter PIN1, the expression of which is induced by mutation of *Pgb2* (Elhiti *et al.*, 2013) or in situations where Pgb2 is retained in the cytoplasm i.e. 35S:Pgb2-GR (A) and (B) lines in the absence of DEX (Supplementary Fig. S5).

A putative function for IAA accumulation is to activate the expression of genes necessary for the acquisition of embryogenic competence (Raghavan, 2004), including *SERK1*, the best characterized marker of embryogenic cells in several systems (Schmidt *et al.*, 1997; Hecht *et al.*, 2001; Somleva *et al.*, 2007). The expression of this gene, sufficient to trigger the formation of apomictic embryos *in vivo* (Albertini *et al.*, 2005) and somatic embryos *in vitro* (Hecht *et al.*, 2001), follows a pattern similar to that of several IAA biosynthetic genes consistent with Pgb2 regulation (Supplementary Fig. S6).

In conclusion, this study demonstrates that the effects of Pgb2 on Arabidopsis somatic embryogenesis are influenced by its cellular localization. Retention of Pgb2 in the cytoplasm, where the protein retains its ability to scavenge NO (Supplementary Fig. S7), phenocopies the effects of *Pgb2* mutation in inducing somatic embryogenesis through suppression of *MYC2* and induction of IAA synthesis and accumulation. These effects are reversed when Pgb2 is translocated into the nucleus. Thus, we conclude that Pgb2 function during Arabidopsis somatic embryogenesis is exercised only when the protein is present in the nucleus and cannot exclude the possibility this might be a universal mechanism in dicotyledonous plants.

## Supplementary Material

Supplementary Data

## Abbreviations:

2,4-D: 2,4-dichlorophenoxyacetic acid
AMI1: amidase 1
cPTIO: 2-(4-carboxyphenyl)-4,4,5,5-tetramethylimidazoline-1-oxyl-3-oxide
CYP79B2: cytochrome P450 CYP79B2
DEX: dexamethasone
DIG: digoxigenin
GR: glucocorticoid receptor
IAA: indole-3-acetic acid
LS: Least Squares
MS: Murashige Skooge
NO: nitric oxide
Pgbs: phytoglobins
Pgb2: phytoglobin 2
SERK1: somatic embryogenesis receptor kinase1
TSB1: tryptophan synthase-α subunit
TSB2: tryptophan synthase-β subunit.
