# Supplementary material for: Cellular localization of the Arabidopsis class 2 phytoglobin influences somatic embryogenesis
Source: J Exp Bot. 2017 Feb 11;68(5):1013–23. doi: 10.1093/jxb/erx003 (PMC5441859; doi:10.1093/jxb/erx003)
Supplement: Supplementary Data [file erx003_Supplementary_Data.zip › supplementary_figures_S1_S7.pdf]

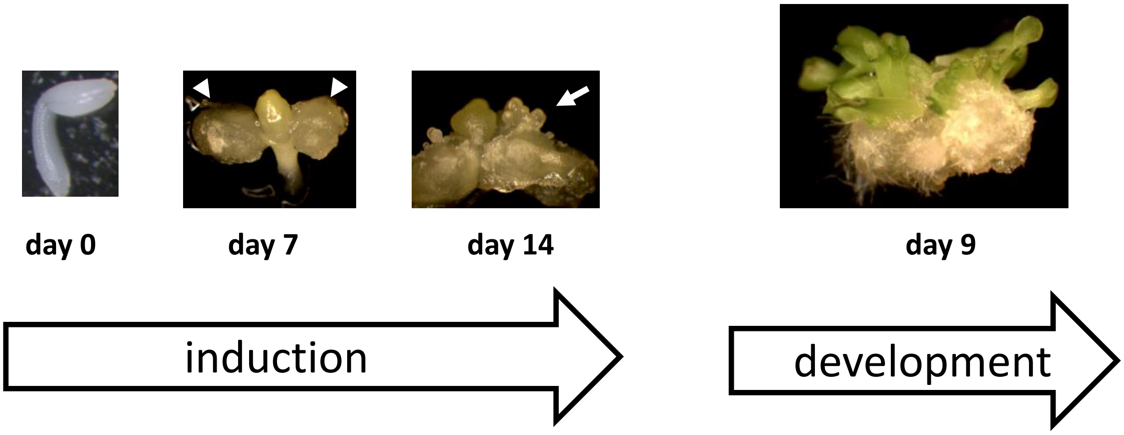

Supplementary Fig. S1. Induction and development of Arabidopsis somatic embryos. Zygotic embryos are cultured on an auxin containing induction medium causing swelling of the cotyledons (arrowheads) and formation of embryogenic tissue (arrow). Somatic embryo growth is completed after 9 days on development medium.

Supplemental Fig. 1

(SalI-YFP-linker-Pgb2-SalI)

TAAGCAGTCGACATGGGCAAGGGCGAGGAGCTGTTCACCGGGGTGGTGCCCATCCTGGTCGAGCTGGACGGCGA
CGTAAACGGCCACAAGTTCAGCGTGTCCGGCGAGGGCGAGGGCGATGCCACCTACGGCAAGCTGACCCTGAAGT
TCATCTGCACCACCGGCAAGCTGCCCGTGCCCTGGCCCACCCTCGTGACCACCTTCGGCTACGGCCTGCAGTGCTT
CGCCCGCTACCCCGACCACATGAAGCAGCACGACTTCTTCAAGTCCGCCATGCCCGAAGGCTACGTCCAGGAGCG
CACCATCTTCTTCAAGGACGACGGCAACTACAAGACCCGCGCCGAGGTGAAGTTCGAGGGCGACACCCTGGTGAA
CCGCATCGAGCTGAAGGGCATCGACTTCAAGGAGGACGGCAACATCCTGGGGCACAAGCTGGAGTACAACTACA
ACAGCCACAACGTCTATATCATGGCCGACAAGCAGAAGAACGGCATCAAGGTGAACTTCAAGATCCGCCACAACA
TCGAGGACGGCAGCGTGCAGCTCGCCGACCACTACCAGCAGAACACCCCCATCGGCGACGGCCCCGTGCTGCTG
CCCGACAACCACTACCTGAGCTACCAGTCCGCCCTGAGCAAAGACCCCAACGAGAAGCGCGATCACATGGTCCTC
CTGGAGTTCGTGACCGCCGCCGGGATCACTCTCGGCATGGACGAGCTGTACAAGCGCAACGCAATGGAGAGATT
GGGTTTACAGAGAAGCAAGAAGCTTTGGTGAAGGAATCGTGGGAGATACTGAAACAAGACATCCCCAAATACAGC
CTTCACTTCTTCTCACAGATACTGGAGATAGCACCAGCAGCAAAAGGCTTGTTCTCTTTCCTAAGAGACTCAGATGA
AGTCCCTCACAACAATCCTAAACTCAAAGCTCATGCTGTTAAAGTCTTCAAGATGACATGTGAAACAGCTATACAGC
TGAGGGAGGAAGGAAAGGTGGTAGTGGCTGACACAACCCTCCAATATTTAGGCTCAATTCATCTCAAAAGCGGCG
TTATTGACCCTCACTTCGAGGTGGTGAAAGAAGCTTTGCTAAGGACATTGAAAGAGGGGTTGGGGGAGAAATACA
ATGAAGAAGTGGAAGGTGCTTGGTCTCAAGCTTATGATCACTTGGCTTTAGCCATCAAGACCGAGATGAAACAAGA
AGAGTCAGTCGACTAAGCA

Supplementary Fig. S2. Sequence of the gBlock fragment SalI-YFP-linker-Pgb2-SalI used to visualize Pgb2.

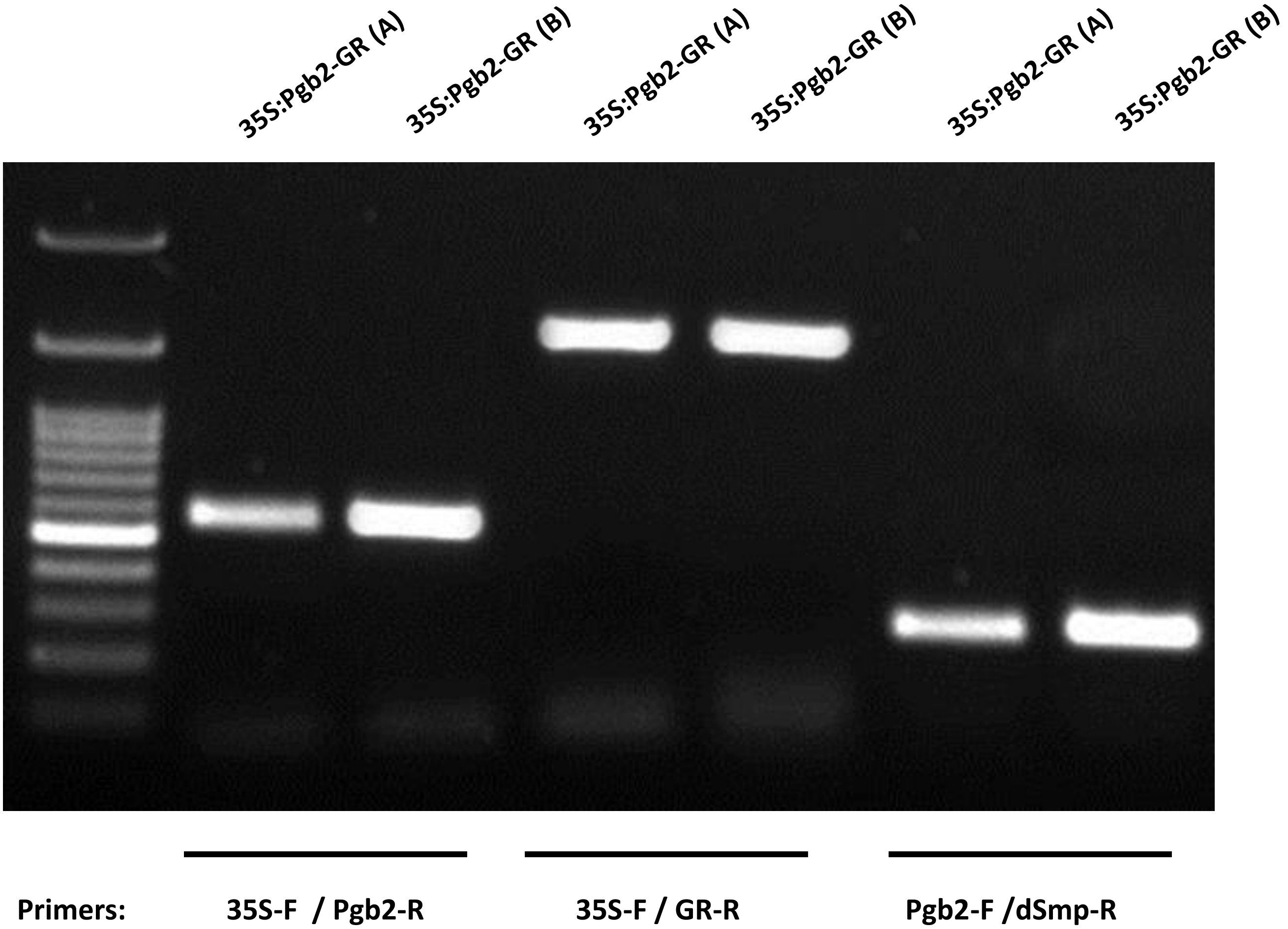

Supplementary Fig. S3. Characterization of the two inducible 35S:Pgb2-GR (A) and (B) lines. PCR analysis of genomic DNA to detect the 35S:Pgb2-GR construct, using primers 35S-F/Pgb2-R and 35SF/GR-R, and to detect the T-DNA insert disrupting Pgb2, using primers Pgb2-F/dSmp-R.

Supplemental Fig. 3

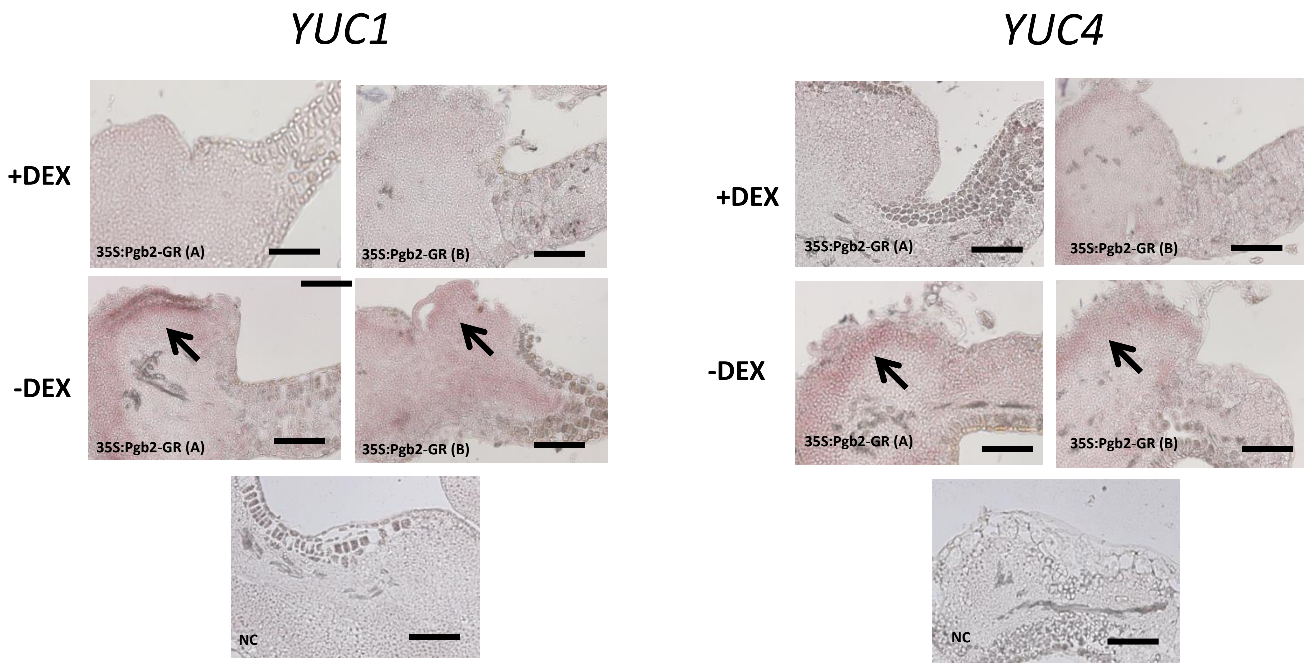

Supplementary Fig. S4. RNA *in situ* hybridization of *YUC1* and *4* during somatic embryogenesis. RNA *in situ* hybridization was carried out at day 7 of induction in expanded cotyledons of zygotic explants of the 35S:Pgb2-GR (A) and (B) lines cultured in the presence or absence of DEX. NC, negative control using sense riboprobes. Scale bar, 150 µm.

Supplemental Fig. 4

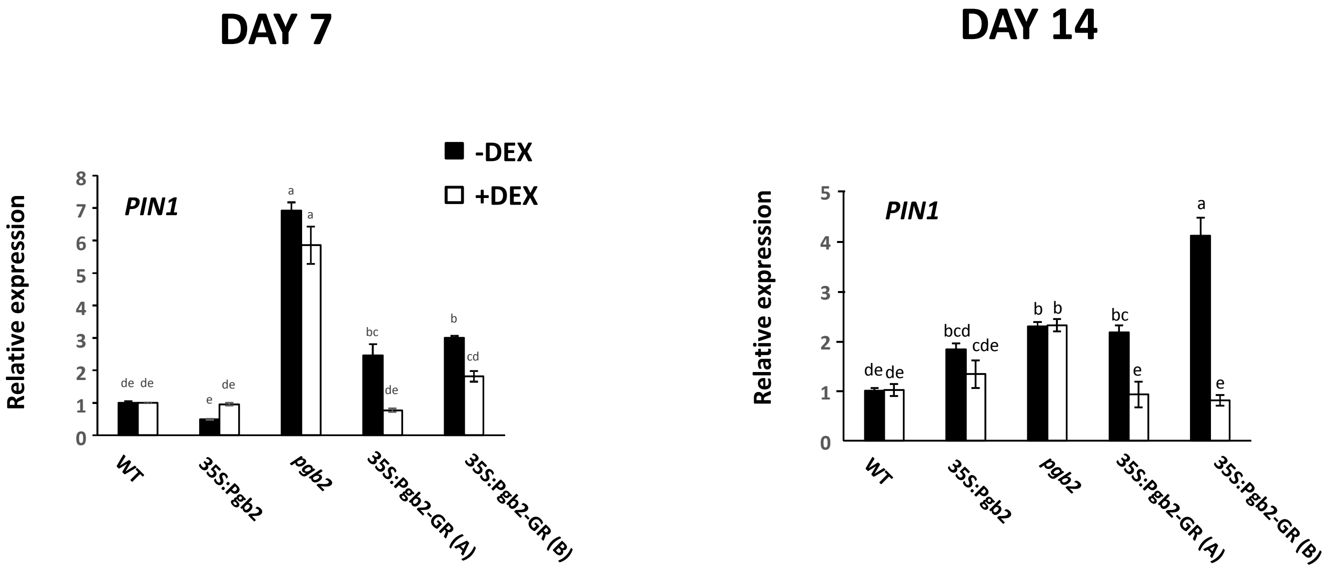

Supplementary Fig. S5. Expression of PIN1 during the induction of somatic embryogenesis. Relative transcript levels of PIN1 at day 7 and 14 of induction. Values±SE are means of three biological replicates and are normalized to the respective wild type values, with or without DEX, set at 1. Letters on bars indicate statistically significant differences, $P<0.05$. Lines utilized: WT, wild type line; 35S:Pgb2, line ectopically expressing *Pgb2*; *pgb2*, line suppressing *Pgb2*; 35S:Pgb2-GR (A) and (B), lines in which Pgb2 tagged to the glucocorticoid receptor (GR) was overexpressed in a *pgb2* background.

Supplemental Fig. 5

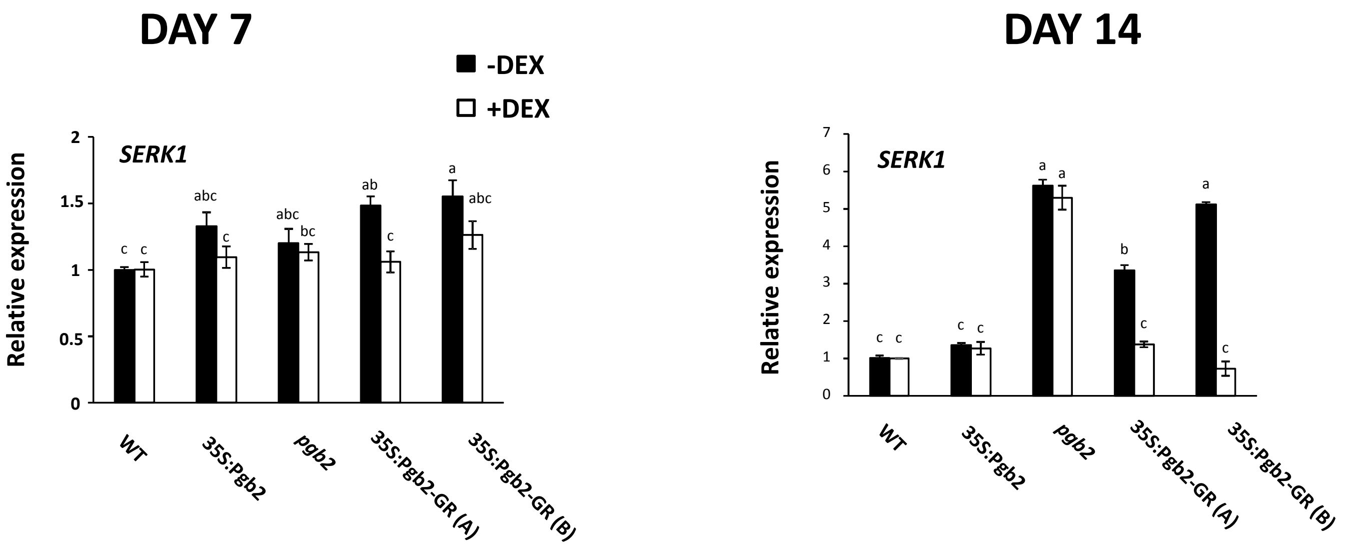

Supplementary Fig. S6. Expression of SERK1 during the induction of somatic embryogenesis. Relative transcript levels of SERK1 at day 7 and 14 of induction. Values±SE are means of three biological replicates and are normalized to the respective wild type values, with or without DEX, set at 1. Letters on bars indicate statistically significant differences, *P*<0.05. Lines utilized: WT, wild type line; 35S:Pgb2, line ectopically expressing *Pgb2*; *pgb2*, line suppressing *Pgb2*; 35S:Pgb2-GR (A) and (B), lines in which Pgb2 tagged to the glucocorticoid receptor (GR) was overexpressed in a *pgb2* background.

Supplemental Fig. 6

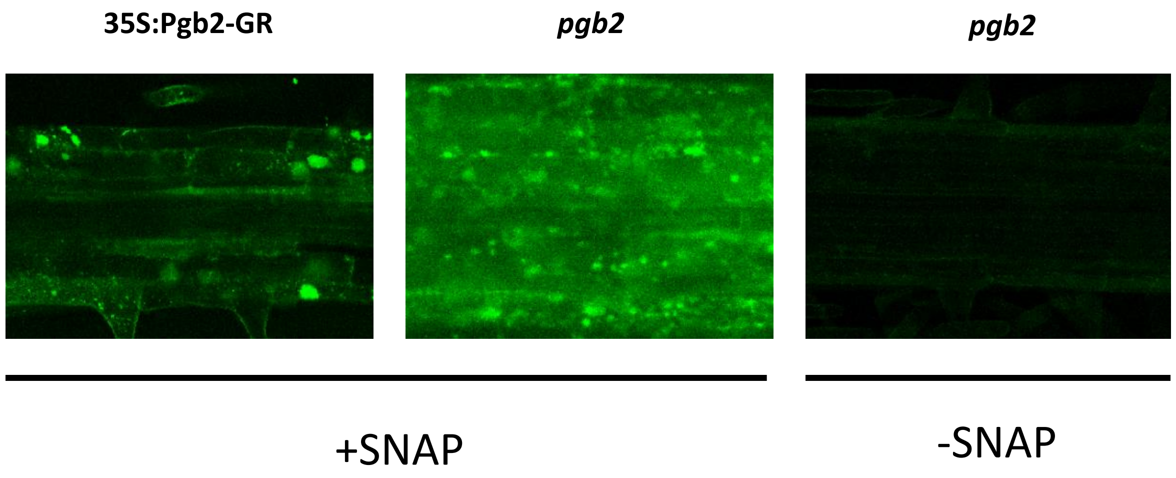

Supplementary Fig. S7. Localization of NO in root cells. NO was visualized using DAF-2DA in the presence of the NO donor S-nitroso-N-acetylpenicillamine (SNAP). Localization studies were performed in the 35S:Pgb2-GR line and the *pgb2* line where Pgb2 is suppressed.

Supplemental Fig. 7
